# Men’s reactions to gender inequality in the workplace: From relative deprivation on behalf of women to collective action

**DOI:** 10.3389/fpsyg.2022.999750

**Published:** 2022-11-17

**Authors:** Silvia Mazzuca, Silvia Moscatelli, Michela Menegatti, Monica Rubini

**Affiliations:** Department of Psychology, Alma Mater Studiorum University of Bologna, Bologna, Italy

**Keywords:** gender inequalities, relative deprivation on behalf of women, collective action, moral conviction, fear, guilt, workplace

## Abstract

Over recent years, the role of men as women’s allies in the struggle for gender equality has become increasingly important. Previous research has shown that often men do not fight gender inequalities as they fail to recognize the severity of discrimination against women (e.g., in hiring). In this study (*N* = 427), we examined whether men who experienced relative deprivation on behalf of women—a form of relative deprivation that stems from the awareness that women hold a less privileged position in society—were more motivated to engage in collective action to support gender equality in the workplace. The findings showed that men’s feelings of deprivation on behalf of women were associated with a greater willingness to engage in collective action for gender equality. This relationship was sequentially mediated by two emotional reactions related to deprivation—increased guilt about gender inequalities and decreased fear of a potential backlash—and the moral conviction of acting for gender equality. These results suggest that men’s awareness of gender inequality at work is an important antecedent to their acting in solidarity with women and that emotions and moral conviction are two psychological processes that turn cognition into behavior. Action to reduce gender inequalities should make men more sensitive to seeing that they hold a privileged position in society and to recognizing the pervasive and harmful nature of women’s deprivation.

## Introduction

“Indeed, gender mainstreaming is an idea whose time has come – for men.”

Michael S. Kimmel, Distinguished Professor of Sociology and Gender Studies at Stony Brook University.

In recent years, gender equality has become an important political goal (Goal 5, 2030 Agenda for Sustainable Development). Till few years ago, this issue did not raise strong public interest, and fighting against unequal treatment of women was implicitly assumed to be a “women’s issue” (European Commission, 2015). For this reason, scientific research was mainly focused on examining whether and how women are motivated to challenge the status quo (e.g., Kelly and Breinlinger, 1995; Yoder et al., 2011). Nevertheless, things have slowly been changing over the last decade, in Western countries at least. Many governments and international institutions have increased their political commitment to eradicating discrimination based on gender (Goertz and Mazur, 2008), and men have started to be more concerned with showing solidarity with women, as can be seen from their increased involvement in movements for gender equality (e.g., HeForShe, Men Advocating Real Change, Token Man, and The Good Lad Initiative). Yet, to date, few studies have examined what motivates men to act to improve women’s rights (e.g., Iyer and Ryan, 2009; Wiley et al., 2012; Stewart, 2017; Radke et al., 2018).

This study aims to expand the theoretical understanding of men’s willingness to engage in collective action for gender equality by investigating whether and under what conditions their awareness of discrimination against women represents an important driver. Given that one of the most pressing issues about gender imbalance is the gender gap in the workplace, with women having consistently lower salaries, lower employment rates, and facing more obstacles to upward mobility than men (World Economic Forum, 2021), we examined men’s perspectives on gender inequality at work and their intentions to collectively act to promote more equal treatment of men and women in that context. The study was conducted in Italy, which is one of the Western countries with the largest gender gap, and where there is a huge need for strategies to promote equal opportunities for women, especially in the labor market (European Institute for Gender Equality (EIGE), 2021).

### Men coping with women’s disadvantage: Relative deprivation on behalf of women

The burden of achieving gender equality has traditionally been placed on women who are usually the main targets of such inequality (Rindfleish and Sheridan, 2003). However, the political solidarity model (Subašić et al., 2008) suggests that social change cannot be achieved without members of the majority acting in solidarity with the minority. Thus, having men collectively act in solidarity with women may be particularly important to achieve gender equality.

Collective action has been a topic of interest to many different social sciences, including psychology, sociology, anthropology, political science, and economics. It commonly refers to any action taken by members of a group—either individually or together—to improve the conditions of their group -in terms of status, power, or influence of the entire group (Wright et al., 1990; see also van Zomeren and Iyer, 2009)—and to achieve a meaningful common objective (Parsons and Shils, 1951; Weber, 1991). Collective action does not necessarily imply being part of a social movement or being an activist. Even if both have social transformation as the aim, social movements operate to bring about social change through collective action—that is social movements rely on collective action, but not all collective action is a social movement (Millward and Takhar, 2019). Similarly, being an activist implies feeling intellectually satisfied and/or reassured by the continued aims of the mobilization, thus by a long-standing engagement in collective action (Millward and Takhar, 2019). Therefore, engagement in a collective action does not automatically imply that an individual is an activist.

Operationally, collective action has been traditionally measured through proxies of behaviors (e.g., favorable attitudes or support or intentions to engage in the action) rather than actual behavior. These proxies have been usually embedded in a group context—for example, “signing a petition to improve the current situation of Blacks in South Africa” (e.g., Cakal et al., 2011; p. 611)—to emphasize that individuals can act on behalf of and for the benefit of a group and that such actions psychologically constitute collective action (e.g., van Zomeren, 2013).

Given that collective action has always been thought of as a driver for social change, most socio-psychological theories and research have focused on the antecedents of disadvantaged group members’ willingness to engage in collective action (e.g., van Zomeren et al., 2008; Stürmer and Simon, 2009; Grant et al., 2015; Radke et al., 2016). Self-identifying as a disadvantaged group member who has been treated unfairly, feeling angry about it, perceiving collective action as an effective way to challenge the status quo, and thinking that a moral principle has been violated, have all been identified as core motivators for collective actions (for a review, see Agostini and van Zomeren, 2021).

However, the reasons underlying collective action by disadvantaged group members may not apply to privileged group members, such as men. Indeed, being part of a privileged group is attractive *per se* (Ellemers et al., 1992; Simon and Hamilton, 1994) and increases social status and positive social identity (Tajfel and Turner, 1986). Consequently, members of these groups may be less motivated to identify with the disadvantaged or to appraise their privileges as illegitimate; thus, they may not experience anger or frustration for the situation of the less-fortunate group members. Moreover, collective actions—especially those that challenge the status quo—may be not directly beneficial for their own group or may even decrease their privileged status (e.g., Branscombe, 1998).

Research on men’s participation in actions that promote female rights and autonomy has found that men are less willing to become engaged in feminist action (i.e., actions that promote female rights) than are women (e.g., Drury and Kaiser, 2014). Indeed, men are less sensitive to gender inequalities (Radke et al., 2018), and are more likely to believe that gains in women’s rights are connected to increased discrimination against men (i.e., they take a zero-sum perspective; Ruthig et al., 2017).

However, when gender inequality is perceived as too pervasive to be ignored, for instance, because it is not limited to a few cases (Iyer and Ryan, 2009), men become more prone to act for the benefit of women. In this situation, they experience the injustice as personally relevant and feel solidarity with women, which in turn motivates them to engage in collective action to compensate (Iyer and Ryan, 2009). In a similar way, Stewart (2017) observed that men low in social dominance orientation, and therefore more supportive of intergroup equality, reported lower levels of hostile sexism toward women, felt more anger about the inequality faced by women and were more likely to engage in collective action against gender inequality than those men who supported hierarchy enhancing ideologies.

Further insights into the understanding of the processes driving men to become more willing to act in solidarity with women to promote gender equality may come from the relative deprivation theory (e.g., Smith and Pettigrew, 2015). This theory is based on the concept that people experience satisfaction or dissatisfaction with their group’s status based on whether its material conditions are consistent with—or lower than—what they expect in comparison with the achievements of other groups or some standard related to one’s group’s past or prospective future situation (e.g., Smith et al., 2012). When individuals perceive that their group is not achieving what it deserves, is discriminated against, or is unfairly disadvantaged compared to another group, they experience feelings of relative deprivation (e.g., Smith et al., 2012); whereas when they perceive that their group is advantaged compared to another less-fortunate group, they experience feelings of relative gratification (e.g., Guimond and Dambrun, 2002).

Although research has shown that for members of the advantaged group the experience of relative gratification is the most common and increases the engagement in collective action aimed at protecting the in-group interests (e.g., Guimond and Dambrun, 2002; Dambrun et al., 2006), members of the advantaged groups may also come to experience a form of deprivation called “relative deprivation on behalf of others” (RDBO), which refers to perceived social injustice faced by an underprivileged outgroup (e.g., Tougas and Beaton, 2002). Experiencing RDBO makes advantaged group members more prone to act for the benefit of the less fortunate group. For example, Beaton and Deveau (2005) found that RDBO increased willingness to act for the benefit of Third-Country people and Leviston et al. (2020) recently observed that RDBO was positively associated with support for multicultural policy among white Australians. Interestingly for the present contribution, RDBO was also found to account for men’s increased intention to support affirmative action programs for women (Veilleux and Tougas, 1989; Tougas and Veilleux, 1990). Drawing on these findings, it seems plausible that men who acknowledge the unfairly disadvantaged position of women in the workplace and therefore experience relative deprivation on behalf of women (hereinafter RDBW) may be more willing to engage in actions that promote more equal treatment of women and men.

### Emotional responses to women’s disadvantage: Fear and guilt

One process through which RDBW may foster men’s willingness to engage in collective actions is through the affective reactions that it evokes. Several studies showed that individuals’ attitudes toward collective action as well as their action tendencies are driven more by their feeling about the group’s situation than by cognitive appraisal alone (e.g., Leach et al., 2002; Smith et al., 2008). For example, feelings of anger or resentment elicited by relative deprivation were found to motivate collective action in the disadvantaged (e.g., Mummendey et al., 1999; van Zomeren et al., 2004).

Less is known about the emotional experience of the advantaged and its role in motivating their intention to take collective action for the benefit of disadvantaged groups. Based on studies concerning relative gratification (e.g., Moscatelli et al., 2014), being aware of one’s group’s more favorable position over outgroups can trigger feelings of fear of losing ingroup advantages, but also guilt, due to recognition that their advantages are based on the unfair treatment of the unfortunate outgroup (Schmitt et al., 2000; Leach et al., 2002).

Fear is an aversive feeling that arises when one perceives a threat or a danger to oneself or to one’s group (Gray, 1989; Öhman, 1993). Research investigating the role of fear in shaping collective action is scarce, with only a few exceptions. Overall, studies showed that fear was associated with avoidance behaviors (e.g., Mackie and Smith, 2002) and that it generally hindered social change (Bar-Tal, 2001). For example, Miller et al. (2009) found that fear suppressed collective action tendencies, even when people were aware of having been mistreated. Similarly, and in a more ecologically valid context, fear was found to be negatively related to support system-challenging collective actions (i.e., actions aimed at changing the status quo; Stefaniak et al., 2020) and collective actions against austerity measures in Greece (Chryssochoou et al., 2013).

Despite such findings, to date, no studies have explicitly considered how fear is related to individuals’ intention to take collective action on behalf of a less-fortunate group, as in the case of men acting to reduce gender-based discrimination. Given that dominant groups experience fear when they face uncertainty about their status in the future (Outten et al., 2012), it seems plausible that men who experience greater relative deprivation on behalf of women would feel lower levels of fear of the potential backlash associated with acting in solidarity with women, which, in turn, might be related to stronger intentions to act for the benefit of women.

Moreover, men confronted with women’s discrimination may come to experience what Hoffman (1976) called “existential guilt,” a feeling that individuals experience in response to their privileged situation compared to another group (Iyer et al., 2003), even for events that one has not directly caused or is not responsible for (Schmitt et al., 2004). Although sometimes guilt can be an immobilizing emotion, it also correlates with the abstract goal of compensation, such as wanting institutions and government to make material compensation, apology, or engage in other forms of systemic restitution to the structurally disadvantaged (Baumeister et al., 1994; Leach et al., 2002).

Interestingly for our purpose, collective guilt was found to be positively associated with higher support for top-down affirmative action in an ethnic-racial group context (Iyer et al., 2003), as well as with greater willingness to engage in, and actual participation in collective actions for the benefit of less-fortunate groups (i.e., non-heterosexuals and Blacks; Mallett et al., 2008). Thus, it seems plausible that men who experience relative deprivation on behalf of women—thus perceiving women as unfairly discriminated—would also experience guilt about women’s unequal treatment and inferior social position in the workplace relative to men’s and, for this reason, that they would be more willing to engage in collective action to promote gender equality.

### Turning emotions into actions: The role of moral conviction

As argued, affective reactions to women’s disadvantaged position may be critical for men’s intention to engage in actions against social injustice. However, the processes through which affective reactions come to be linked to individuals’ action tendencies are still unclear. Research suggested that the influence of emotions on behavior should be considered in relation to motivation (e.g., Chiew and Braver, 2011). Although motivation and emotion are highly related constructs within the domain of affect, motivations are specific, relatively deliberate, and associated with a particular goal, whereas emotions are produced by multiple contingencies, are more impulsive, and are not closely linked to a specific purpose. While emotion reflects an act of “liking/disliking,” motivation signals a state of “wanting/not wanting” (Berridge, 1996). As Carver (2006) suggested, affect emerges when there is a difference between one’s present status and one’s goal state, while motivation develops from this affect and leads to goal re-prioritization to maximize goal fulfillment. Starting from these considerations, we wondered what kind of motivation may be influenced by emotions and mobilize men to end gender inequality.

Research on collective action has recently emphasized the role of moral conviction, namely a strong moral belief about an issue, as one of the most powerful motivators for engagement in collective actions (e.g., van Stekelenburg et al., 2009; Jost et al., 2017; Milesi and Alberici, 2018; Sabucedo et al., 2018). When people hold a moral conviction about an issue (e.g., for example, when they believe that gender equality is part of their moral values) and this conviction is threatened (e.g., a government justifies the legitimacy of gender inequalities), they tend to reaffirm their threatened conviction by expressing stronger intentions to engage in collective actions (e.g., van Zomeren et al., 2011; van Zomeren, 2013). Moral convictions might also overpower the detrimental effects of system justification (i.e., the belief that existing social, economic, and political arrangements are fair; Jost and Banaji, 1994) on the collective action intentions of deprived group members. Considering feminist collective action, De Cristofaro et al. (2021) found that women who held stronger moral convictions against gender inequality were more willing to engage in collective action regardless of the strength of their system justification beliefs.

However, holding a moral principle may not be sufficient to mobilize individuals, especially when this conviction is not threatened. In such a situation, it is important that individuals “moralize” a specific behavior related to that principle; that is they must hold a moral conviction about engaging in that behavior. The extent to which people moralize an action increases their commitment and engagement in that action (e.g., the so-called moral mandate effect, Skitka, 2002; Skitka and Mullen, 2002). Given that recent theorizing in moral psychology suggested that emotions constitute the “motivational” basis for morality and moral behavior (Eisenberg, 2000; Hoffman, 2000; Tangney and Dearing, 2002), we reasoned that emotions might influence the willingness to engage in collective behaviors by altering individuals’ moral motivations, specifically the strength of their moral conviction associated to engaging in a given action. By drawing one’s attention to the morally salient features of the environment, emotions trigger distinctively moral evaluations (Huebner et al., 2009; Feinberg et al., 2019) and moral judgments (Haidt, 2001) and, through these processes, motivate moral behavior and political engagement (Snow and Soule, 2010).

Applying these considerations to the present study, it seems reasonable that men holding a stronger moral conviction about acting for gender equality would be more prone to mobilize. Similarly, the strength of this conviction might be differently related to the emotional reactions of guilt and fear triggered by RDBW, with guilt increasing and fear decreasing such conviction.

### The present research

This research aimed to understand which processes may account for men’s willingness to engage in collective action for gender equality. We examined whether relative deprivation on behalf of women—stemming from men’s awareness of gender inequalities in the workplace—was positively associated with men’s intentions to act to promote more equal treatment between women and men at work. We also analyzed whether this association was sequentially mediated by men’s emotional reactions to their awareness of such inequalities (in terms of guilt and fear) and moral conviction related to engaging in collective solidarity action.

Based on previous evidence showing that experiencing relative deprivation on behalf of a less-fortunate group motivates support for that group (e.g., Leviston et al., 2020), we hypothesized that men’s experience of deprivation on behalf of women would be associated with a stronger intention to act collectively to promote more gender equality (hypothesis 1). We also hypothesized that this effect would be mediated by the emotional reactions of guilt and fear. Specifically, we expected that men’s levels of RDBW would be associated with stronger feelings of existential guilt about women’s inferior position relative to men’s (hypothesis 2a) and with lower feelings of fear of a potential backlash related to fighting against gender-based discrimination (hypothesis 2b). We also expected that feelings of guilt would be related to a stronger moral conviction about acting to promote gender equality (hypothesis 3a), whereas the pattern should be reversed with respect to feelings of fear (hypothesis 3b). We further predicted that the strength of moral conviction about acting to increase social justice would be associated with stronger willingness to engage in collective action on the part of men (hypothesis 4). Overall, we expected that emotions and moral conviction about acting for gender equality would sequentially mediate the relationship between men’s relative deprivation on behalf of women and intentions to engage in collective action to end gender-based discrimination in the workplace (hypothesis 5).

## Materials and methods

### Participants and procedure

Data was collected in September–November 2021 in Italy using the Qualtrics platform. The Bioethical Committee of the University of Bologna approved the research. All participants were assured about the anonymity of the data and provided informed consent before filling in the questionnaire. Participants employed about 15 min to fill in the measures.

The sample size for this study was determined following Fritz and MacKinnon (2007), according to whom 400 participants are sufficient to detect small/medium indirect effects in (complex) mediation, assuming an alpha of 0.05 and a power of 0.80. Additionally, we tested the indirect effects with a bootstrapped confidence interval (e.g., Zhang, 2014). This method does not assume a normal distribution for all the paths and allows the researcher to generate a sampling distribution for the indirect effects empirically. We set a bootstrap of 5,000 to create a sampling distribution with a confidence interval of 95%. If this confidence interval did not include 0, then one would reject the null hypothesis (Schoemann et al., 2017).

Four hundred forty-eight participants were recruited *via* personal social networks (e.g., Facebook, Twitter) of the researchers involved in the study and snowball sampling (every person was asked to send the questionnaire to three persons).

The questionnaire began by asking participants their opinion about the current situation of gender inequalities in the workplace, specifically about women’s discrimination, and to express their feelings about such situation. Then participants were invited to indicate whether they are in favor or against acting to promote more gender equality practices in the workplace and to rate the extent to which such attitude is part of their moral convictions. Given that we were interested in examining variations in the strength of moral conviction associated with engaging in collective action, rather than evaluating the impact of being in favor or against it, we excluded 21 participants who indicated that they were against acting to promote more gender equality (by selecting 1 = completely against, 2 = against, 3 = quite against) or uncertain about it (by selecting 4 = not against nor in favor). At the end of the questionnaire, participants were invited to rate their willingness to engage in a series of collective actions to promote equal treatment between women and men in the workplace.

The final sample consisted of 427 male participants (*M*_age_ = 42.97, SD_age_ = 13.35, range = 18–70), of Italian nationality (81.5% workers; 13.8% university students, 4.7% other). For structural equation modeling (SEM) incorporating latent variables, 5:1 is the commonly recommended observation ratio to estimated parameters (Kelloway, 2015). Our model had 75 parameters, so our sample size can be considered adequate.

### Measures

Unless stated otherwise, all responses were given on a 7-point scale (1 = *not at all*; 7 = *very much*). To measure relative deprivation on behalf of women, three items asked participants to indicate the extent to which, in their opinion, women are “unfairly disadvantaged/discriminated/penalized in the workplace compared to men” (items derived from Leviston et al., 2020; *α* = 0.94).

Emotions were assessed by asking participants to indicate the extent to which they felt a series of feelings when thinking about gender inequalities (“When thinking about gender inequalities in the workplace, as a man, I feel…”). Five items assessed existential guilt: “guilt,” “responsible,” “ashamed,” “embarrassment,” and “discomfort.”^1^ and three fear of a potential backlash associated with achieving more gender equality “fear,” “scared,” “worried.” The order of the emotions was randomized. A principal-axis factor analysis with Oblimin rotation confirmed a 2-factor solution (eigenvalue >1), which accounted for 70.29% of the common variance. The item “responsible” was removed due to low factor loading. The first factor was saturated by the items of existential guilt (*α* = 0.87), and the second factor was indicated by the fear items (*α* = 0.86).

Afterward, participants were invited to rate the extent to which they were in favor or against acting to improve the condition of women at work (1 = *completely against*, 7 = *completely in favor*). As mentioned, only data of participants with a favorable attitude were considered in the analyses (i.e., responses ranging from 5 to 7; see van Zomeren et al., 2011). Participants then indicated the extent to which this attitude was part of their moral conviction on five items derived from Skitka et al. (2021; e.g., “To what extent acting for gender equality is part of your moral conviction?”; *α* = 0.90). We adapted Tausch et al. (2015) scale to measure intentions to engage in collective action. Participants were asked to indicate the extent to which they would have been willing to engage in five actions to promote gender equality at work (e.g., “public demonstration or flash mob;” “sign a petition;” *α* = 0.86).

Given that previous studies have found political orientation to be related to system-justifying ideologies that might hinder social change (e.g., Jost et al., 2017), with right-wing-oriented individuals endorsing system-justifying ideologies to a larger extent than left-wing-oriented individuals (e.g., Kutlaca et al., 2016), we also measured participants political orientation on a 7-point scale ranging from 1 = *extreme left-win*g to 7 = *extreme right-wing.*

## Results

### Preliminary analyses

Table 1 reports the descriptive statistics, the measures of skewness and kurtosis that have been used to determine if indicators met normality assumptions (Kline, 2005), and the correlations among all variables. Overall, the skewness indexes indicate that all the study variables were moderately skewed. In contrast, kurtosis indexes indicated that variables’ distributions were similar to a normal distribution, except for RDBW and fear, which were more heavy-tailed, and age, which was less heavy-tailed than the normal distribution. Given that values of skewness fall between –3 and + 3, and kurtosis is appropriate from a range of –10 to +10 when utilizing SEM (Brown, 2006), our indexes satisfied the assumption of normality.

**Table 1:** Descriptive statistics and correlations between study variables.

Measures	*M*	SD	Skew	Kurt	2	3	4	5	6	7
1. RDBW	4.97	1.19	−0.98	1.4	0.397***	−0.336***	0.450***	0.462***	−0.255***	−0.083
2. Guilt	3.51	1.54	−0.11	−0.86		−0.209***	0.413***	0.486***	−0.111*	0.055
3. Fear	1.94	1.12	1.4	1.84			−0.400***	−0.259***	0.250***	−0.115*
4. Moral conviction	5.52	1.07	−0.78	0.94				0.539***	−0.262***	0.082
5. Collective action	3.85	1.37	−0.24	0.47					−0.292***	0.028
6. Political orientation	2.77	1.51	0.7	−0.12						−0.079
7. Age	42.97	13.35	−0.33	−1.07						

RDBW = Relative deprivation on behalf of women; ***p < 0.001; *p < 0.05.

With regards to correlations, overall, men’s experience of relative deprivation on behalf of women was positively correlated with their willingness to engage in collective action to increase gender equality and feelings of guilt, whereas it was negatively related with feelings of fear. RDBW was also positively correlated with moral conviction about acting for gender equality. Guilt was positively associated with moral conviction about acting for gender equality and willingness to engage in collective action, which instead were both negatively correlated with fear. Interestingly, political orientation was negatively associated with all study variables except for fear, with which it had a positive relationship. In other words, men who were more right-wing oriented experienced lower RDBW, lower guilt, and stronger fear; they also reported lower moral conviction about acting for gender equality and lower intentions to engage in collective action than those who were more left-wing oriented. Thus, we included political orientation as a covariate in the main analyses.

Surprisingly, age did not correlate significantly with any of the study variables except for fear, with older men experiencing lower fear than younger men. Given these results, we did not include age as a covariate in our analysis.

### Mediational analyses

To test our hypotheses, we estimated a model in which RDBW was included as a predictor, fear and guilt as parallel first mediators, moral conviction as second mediator, and intentions to engage in collective action as the outcome variable. All variables were latent variables, with items used as indicators. To adjust for measurement error, SEM with latent variables (Bollen, 1989) was performed using Mplus version 8.3 (Muthén and Muthén, 2019). Model parameters were estimated using the maximum likelihood (ML) method. To test for mediation, bootstrap (5,000 resamples) estimates of indirect effects and bootstrapping bias-corrected confidence intervals (Cis) were calculated. We evaluated the model fit by means of multiple indices: the Comparative Fit Index (CFI) and the Tucker-Lewis Index (TLI), with values higher than 0.90 indicative of an acceptable fit and values higher than 0.95 suggesting an excellent fit; and the Root Mean Square Error of Approximation (RMSEA), with values below 0.08 indicative of an acceptable fit and values lower than 0.05 representing a very good fit (Byrne, 2012). In addition, we inspected the 90% confidence interval of the RMSEA: when the upper bound of this confidence interval is ≤0.10, the model fit can be considered acceptable (Chen et al., 2008).

Previous evidence on the relations between cognitive appraisals and emotions for collective behaviors (e.g., Smith et al., 2012) and moral convictions (e.g., Wisneski et al., 2020; D’Amore et al., 2021) suggests that we cannot exclude that RDBW maintains a direct effect on moral conviction or intentions to engage in collective action. Thus, we first estimated a partially mediated model. For the sake of completeness, we also estimated the fully mediated and the non-mediated model.

As shown in Table 2, all the estimated models fit the data reasonably. The comparative fit index (CFI) and the Tucker-Lewis index (TLI) exceeded 0.95, and the SRMR and the RMSEA were less than 0.08. However, the non-mediated and fully mediated models provided worse fits to the data than the partially mediated model. Thus, we might conclude that the partially mediated model is the best and most parsimonious model.

**Table 2:** Fit indices for the three models.

Model	*χ* ^2^	df	CFI	TLI	RMSEA	SRMR	AIC	BIC
Fully mediated	356.744***	178	0.968	0.968	0.049	0.064	25,335.7	25,627.6
Part. mediated	302.088***	175	0.977	0.972	0.041	0.036	25,287.1	25,591.2
Non mediated	361.416***	178	0.967	0.961	0.049	0.072	25,340	25,632.3

***p < 0.001.

**Figure 1:**
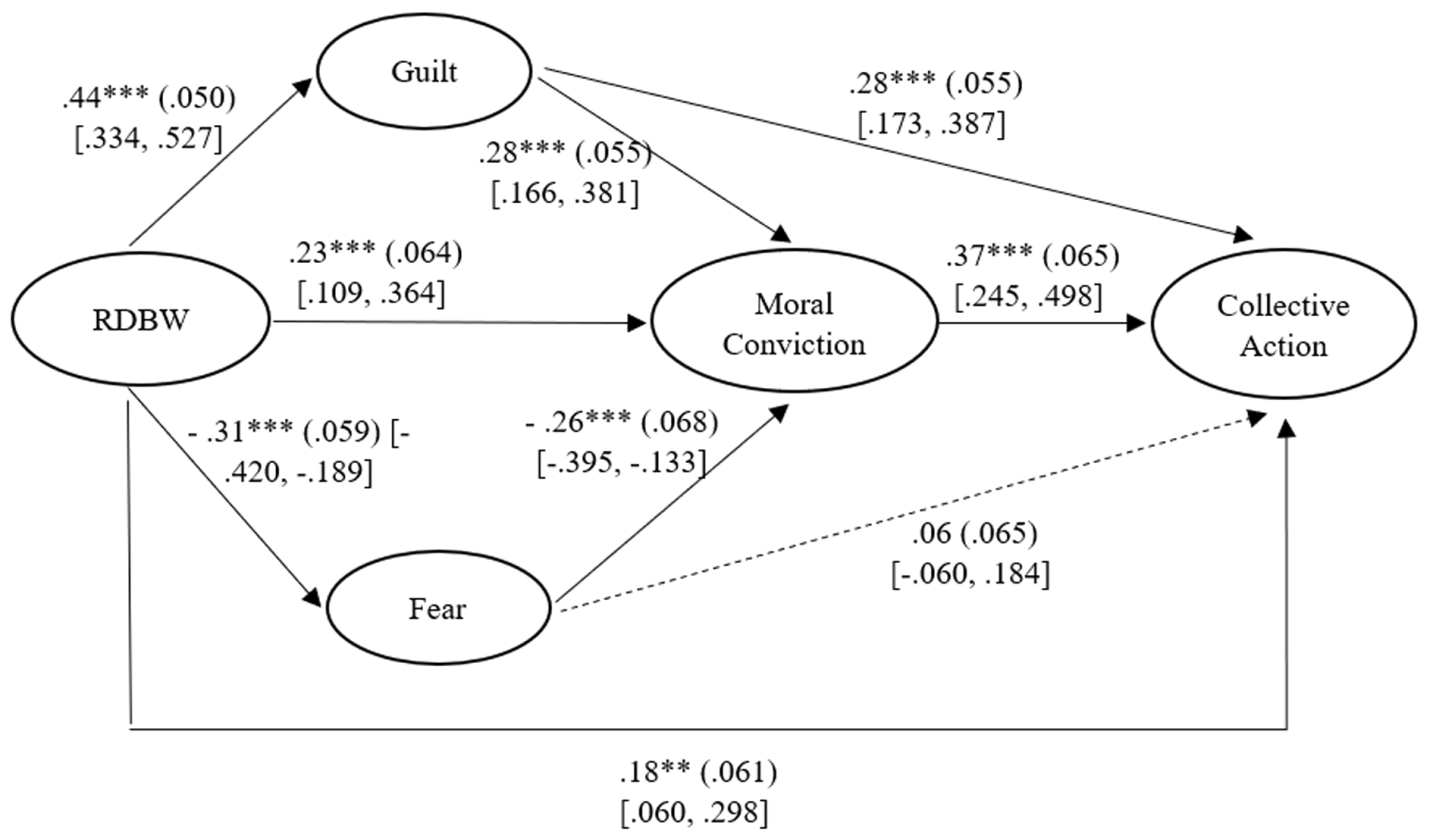
Standardized solution of the model testing the relations among RDBW (relative deprivation on behalf of women), emotions, moral conviction, and collective action, with *β* (SE) and [95% CI]. Political orientation was included in the model as covariate, but it is not shown in the figure. **p < 0.01. ***p < 0.001.

Experiencing RDBW was associated with greater willingness to become more active in challenging gender-based discrimination (hypothesis 1). As shown in Figure 1, RDBW was related to a higher experience of existential guilt about discrimination against women (hypothesis 2a) and a lower experience of fear of potential backlash associated with reducing such discrimination (hypothesis 2b). These feelings of guilt and fear were significantly related to moral conviction, respectively in a positive and a negative way (hypotheses 3a and 3b). Finally, moral conviction about acting to attain more gender equality was associated with stronger intentions to engage in collective action aimed at promoting more equal treatment of women and men in the workplace (hypothesis 4).

The inspection of indirect effects (Table 3) supported the expected paths and revealed that emotions and moral conviction sequentially mediated the relation between RDBW and collective action intentions (hypothesis 5). Interestingly, the relation between fear and collective action was fully mediated by moral conviction, whereas the association between guilt and collective action intentions was partially mediated.

**Table 3:** Total, total indirect, and specific indirect effects of relative deprivation on behalf of women (RDBW) on Collective action.

Effects of RDBW on Collective Action	Estimate (SE)	[95% CI]
Total	0.45 (0.050)	[0.347, 0.541]
Total indirect	0.27 (0.039)	[0.191, 0.344]
RDBW → fear→ collective action	−0.02 (0.020)	[−0.059, 0.020]
RDBW → guilt→ collective action	0.12 (0.029)	[0.070, 0.184]
RDBW → moral conviction→ collective actions	0.09 (0.028)	[0.038, 0.146]
RDBW →fear→ moral conviction→ collective action	0.03 (0.012)	[0.012, 0.056]
RDBW→ guilt→ moral conviction→ collective action	0.05 (0.012)	[0.024, 0.069]

With regards to the covariate, political orientation was negatively related to RDBW, *β* = −0.26, SE = 0.048, *p* < 0.001, 95% CI [−0.347, −0.164], moral conviction, *β* = −0.10, SE = 0.045, *p* = 0.023, 95% CI [−0.190, −0.015], and collective action *β* = −0.14, SE = 0.048, *p* = 0.003, 95% CI [−0.239, −0.050]. Political orientation was not significantly related to guilt, *β* = −0.02, SE = 0.050, *p* = 0.635, 95% CI [−0.116, 0.065], whereas it had a positive association with fear, *β* = 0.20, SE = 0.053, *p* < 0.001, 95% CI [0.102, 0.309]. In other words, the more participants were right-wing oriented, the lower their levels of RDBW, moral conviction, and collective action intentions were, and the higher their feelings of fear^2^.

## Discussion

This research aimed to examine the factors that might motivate men to engage in collective action for gender equality. To this end, we tested whether the experience of relative deprivation on behalf of women, intended as the awareness that women suffer an unjust disadvantage in the workplace compared to men, was associated with a greater tendency for men to act in solidarity with women to end gender-based discrimination. We also analyzed whether the relation between RDBW and intentions to engage in collective action was sequentially mediated by men’s affective reactions to their awareness of women’s disadvantage—in terms of increased guilt and reduced fear—and by moral conviction associated with acting to promote gender equality.

Overall, our findings highlighted that men’s awareness of women’s inferior treatment and social position relative to men’s in the workplace is an important antecedent for their engaging in the fight against gender inequality, providing support for the hypothesized patterns. First, we found a direct and positive association between RDBW and men’s willingness to engage in collective action. Second, we observed that RDBW was positively associated with feelings of existential guilt about the different treatment of women and men at work, while being negatively related to feelings of fear of a potential backlash that may derive from acting in solidarity with women. In turn, these emotions played a different role in the relationship between RDBW and the intention to act collectively. Whereas fear was associated with lower levels of moral conviction about the importance of acting to promote more gender-equal employment practices, guilt was related to a stronger moral conviction, which in turn strengthened men’s activism.

Taken together, these findings seem to suggest that men’s awareness of discrimination suffered by women in the workplace—embedded in the concept of RBDW—may represent a critical incentive for men’s willingness to act in solidarity with women to reduce such discrimination, by influencing men’s emotional experiences, and by altering their moral convictions about acting for gender equality. Men’s sensitivity to women’s disadvantaged position may be particularly critical to consider because it influences those emotions that might otherwise be counter-productive to action, such as fear, while holding men’s moral compass about acting for equality. In other words, this experience of deprivation seems to “reassure” men that being allies of women does not mean losing their privileges, and that cooperating in an ongoing effort to attain equality might benefit both women and men.

Focusing on the experience of relative deprivation on behalf of a less fortunate group (such as women compared to men) may therefore be vital to a further understanding of what motivates members of advantaged groups to act collectively to challenge social inequalities. Failing to recognize the severity of discrimination against women in the workplace may lead men to underestimate the socio-psychological effects of such discrimination and, consequently, to be unlikely to act as allies. Raising men’s awareness may be even more critical since gender biases are not always explicit and overtly hostile and can sometimes appear to be even “justified” (e.g., Glick and Fiske, 2011; Moscatelli et al., 2021).

Consequently, the more men become aware of such gender inequalities, the more they may overcome the barriers experienced by those men who are less prone to confront discrimination and thus challenge them. Allies generally incur fewer adverse reactions when they draw attention to prejudice than do members of the targeted group who take the same actions (Rasinski and Czopp, 2010). As such, male allies may be perceived as acting more legitimately or appropriately by other men and be therefore effective in raising and motivating other men’s allyship.

### Theoretical and practical implications

This study contributes in several ways to theorization concerning advantaged group members’ motivation to engage in collective action aimed at challenging social inequality (e.g., Grant et al., 2015; Leviston et al., 2020; Agostini and van Zomeren, 2021). First, it highlighted that the advantaged group’s cognitive appraisal and acknowledgment of discrimination against a disadvantaged group (i.e., relative deprivation on behalf of others) can act as a driver of engagement in collective actions that can benefit the disadvantaged group (e.g., Leviston et al., 2020). Similarly, it showed that the emotional experience associated with such appraisal as well as individuals’ moral conviction about the importance of engaging in collective action both motivate the advantaged to challenge social inequalities (e.g., Smith et al., 2012) even in the context of gender-based discrimination in the workplace.

These results add to the literature on the relationship between relative deprivation and collective action (e.g., Smith et al., 2012) by showing that it can be accounted for by two sequential processes: (a) the emotional reaction triggered by this experience of deprivation, namely existential guilt and fear and (b) the moral motivation fostered by such emotions that determine the strength of moral conviction that advantaged group members associate to engaging in a specific action. Considering the emotional processes that link cognition with behavior, we found that the perception of unfair outgroup disadvantage, and thus ingroup advantage, was positively related to guilt and negatively associated with fear, which were differently associated with action intentions. Whereas men’s experience of guilt was positively associated with a greater willingness to engage in actions directed towards the specific goal of reducing gender inequality in the workplace, their experience of fear inhibited these intentions. This evidence adds to prior work on existential guilt (e.g., Baumeister et al., 1994; Schmitt et al., 2004) and suggests that guilt might be a relatively strong predictor of compensation of social inequality not only at the abstract level (e.g., making amends; Tangney et al., 1996) but also at more concrete, behavioral level.

Concerning fear, the results go further than previous findings (e.g., Miller et al., 2009; Moscatelli et al., 2014) and reveal that members of advantaged groups might refrain from joining the fight for intergroup equality out of the specific fear of potential negative consequences that altruistic actions can have for themselves (for example, by reducing their privileges). Interestingly, our results further highlight that experiencing relative deprivation on behalf of a less-fortunate group can decrease this fear. This might imply that relative deprivation on behalf of others might more generally lessen feelings of threat due to a potential backlash associated with actions that may benefit a less-fortunate outgroup. For example, future research could explore whether RDBW reduces gender-based zero-sum thinking and beliefs (e.g., Kuchynka et al., 2018; Kosakowska-Berezecka et al., 2020), which are known to foster workplace gender biases and opposition to gender equity.

Our results also point out for the first time that the emotional experience triggered by the deprivation on behalf of women affected the extent to which men were morally concerned about fighting for women’s equal opportunities. While the experience of guilt increased men’s moral conviction about acting for gender equality, fear decreased it. Thus, guilt appears to motivate men to engage in specific actions by increasing the moral importance attributed to those actions; fear seems instead to drive towards an opposite pattern, thus de-motivating men (for similar reasoning about how emotions motivate avoidance-approach orientation, see Leach et al., 2002; Mackie and Smith, 2002).

These results suggest that regulating one’s moral compass is a process by which collective guilt and fear are associated with collective action for the benefit of the disadvantaged group. In this respect, one might argue that the moralization of an action—intended as the variation in the strength of one’s moral conviction associated with engaging in an action (e.g., Skitka et al., 2021)—may represent a way through which members of privileged groups “cleanse” their moral standing from the negative image made salient by the experience of relative deprivation on behalf of the disadvantaged. Individuals are motivated to see themselves and their group as good and moral (e.g., Ellemers et al., 2013; Moscatelli et al., 2019), and perceiving that the ingroup is unfairly advantaged compared to an outgroup may lead them to experience ethical dissonance (Feinberg et al., 2019), a psychological tension that threatens one’s moral image. In turn, this dissonance may lead privileged group members to act to benefit the structurally disadvantaged group (Leach et al., 2002) to reduce this tension and cleanse their morality. By expressing a high willingness to engage in collective action for gender equality, men might try to increase their social and moral acceptability. Of course, future research might explore this possibility, either by considering different kinds of action or looking at actual behaviors. Although behavioral intentions may be conceptually closer and more accurate predictors of actual behaviors than behavioral measures (e.g., Kim and Hunter, 1993; Cooke et al., 2016; Hamilton et al., 2020), individuals may be “satisfied” that they have done enough by expressing their willingness to act and therefore may be less likely to deepen their engagement with that cause through actual behavior (a phenomenon called “slacktivism;” see Schumann and Klein, 2015).

Overall, these results lend support to more general theoretical models on the relationship between cognition, emotion, and motivation by highlighting the intimate and closely interacting nature of these processes for behaviors (Carver, 2006). In this respect, a novelty of the present study is that it addressed the lack of a motivational element in previous explanations of collective action (Agostini and van Zomeren, 2021) by integrating moral conviction with previous motives and by explicitly considering the moral conviction related to action in term of a general abstract principle. It is also important to note that these findings encompass a chain of effects linking cognition to action, thus validating what was stated by Fiske (1992) that “thinking is for doing.” However, at the end of doing the thinking is not enough. As Kruglanski et al. (2020) claimed, “all thinking is wishful thinking,” meaning that thinking goes hand in hand with emotions and motivations, as affective states and desires come into play to support the one side’s cognition and the other side’s action.

More generally, this study contributes to an understanding of the factors that might lead men to act as allies of women. Previous research on this issue revealed that factors related to identity, ideologies, and norms could facilitate men’s allyship with women (Iyer and Ryan, 2009; Wiley et al., 2012; Stewart, 2017; Radke et al., 2018; Menegatti et al., 2022). This study tries to identify a general process model by considering possible variables that might inhibit or enhance men’s intentions to engage in collective action for gender equality.

At the practical level, the solidarity movement for gender equality should therefore make men more sensitive to detecting and acknowledging the injustice of their privileged position in society and recognizing the pervasive and harmful nature of women’s experience of deprivation and its consequences. This implies changing men’s perceptions and beliefs about the prevalence and nature of both overt and subtle discrimination against women in the workplace (and in society). For example, encouraging men to reject status-legitimizing beliefs (Swim et al., 2001, 2004; Hyers, 2007) and zero-sum beliefs (e.g., Kosakowska-Berezecka et al., 2020) will make them more likely to acknowledge the unfair deprivation of women and maybe more prone to confront gender inequalities.

### Limitations and future directions

The present study is not without limitations. First, whereas self-report measures are advantageous in terms of administration, self-presentation and attribution response biases can limit their validity (Robinson and Clore, 2002), as men can refrain from giving answers overtly against gender equality. Future research might therefore use more implicit measures to test our hypotheses.

It would also be essential to employ measures that capture concrete behaviors instead of (or in addition to) behavioral intentions and differentiate among different kinds of behaviors. For example, men may be asked to report the daily frequency of their behaviors related to gender equality, to choose a behavior using a game theory paradigm, or to report the extent to which they would be willing to carry out active vs. passive facilitation behaviors at work (e.g., Cuddy et al., 2007) towards a woman. This will allow extending the validity of the model proposed in this study to actual behaviors and overcoming the methodological limits of self-report measures.

Similarly, it would also be important that future studies consider other types and contexts of gender inequality in addition to the one considered in the study. For example, it would be interesting to investigate men’s awareness of gender imbalance in leadership and political participation. Unbalance in roles and duties within close relationships, as well as disparities in education might be further critical domains to investigate in order to counteract gender inequality.

Second, since this study had a cross-sectional, correlational design, the directionality implied in our analyses is assumed. Future research should include experimental manipulation and test the variability in emotions and levels of moral conviction when different scenarios of gender inequalities (that should induce RDBW) are presented.^3^

Third, to obtain a deeper insight into the processes linking relative deprivation on behalf of others and collective action intentions, for future work it would be necessary to include other negative emotions—such as anger or pride—which have been found to influence collective actions (e.g., Leach et al., 2002; Harth et al., 2008; Panno et al., 2022), and that may be differently associated with a moral motivation to act. Finally, although we specifically considered the moral conviction associated with action instead of in terms of a general abstract principle, the importance of the latter for individuals’ collective behaviors (e.g., Agostini and van Zomeren, 2021) suggests that future studies should implement both perspectives within the same study. This is very important in order to disentangle their role in influencing individuals’ behaviors.

## Conclusion

Many past efforts to increase gender equality stemmed from women’s activism. However, critical challenges remain in some key areas, such as work, and the contribution of men as allies cannot be ignored. The next “big thing” in gender equality would be to include men as a target of action. Although increasing gender-sensitive macro-economic policies is of crucial importance to supporting women’s empowerment, our study indicates that men’s awareness of gender inequalities is an important factor in “getting men on board” so that they can serve as allies of women. Although previous studies have shown that men are generally motivated to maintain male power in the workplace through subtle ways (e.g., Rubini and Menegatti, 2014), our study suggests that today men have more accessible knowledge about the unfairness of their privileges, and this makes them more “ready” to act in solidarity with women to achieve more gender equality.

## Data availability statement

The dataset presented in this study can be found in online repository at: https://osf.io/q7kjs/.

## Ethics statement

The studies involving human participants were reviewed and approved by Bioethical Committee of the University of Bologna. The participants provided their written informed consent to participate in this study.

## Author contributions

SMa and SMo collected the data. SMa conducted the statistical analyses. SMa and SMo wrote the first draft of the manuscript. All authors conceived and designed the study. All authors contributed to the article and approved the submitted version.

## Funding

This work was supported by the Italian Ministry of Education, University and Research (MIUR), PRIN grant to Monica Rubini entitled “The Psychology of Economic Inequality” Prot. 2017924l2b.

## Conflict of interest

The authors declare that the research was conducted in the absence of any commercial or financial relationships that could be construed as a potential conflict of interest.

## Publisher’s note

All claims expressed in this article are solely those of the authors and do not necessarily represent those of their affiliated organizations, or those of the publisher, the editors and the reviewers. Any product that may be evaluated in this article, or claim that may be made by its manufacturer, is not guaranteed or endorsed by the publisher.

## Supplementary material

The Supplementary material for this article can be found online at: https://www.frontiersin.org/articles/10.3389/fpsyg.2022.999750/full#supplementary-material

## Footnotes

^1^Following Leach et al. (2006), we included 3 items related to shame (shame, embarrassment, discomfort) because Italian speakers often use guilt and shame interchangeably. However, the concept of shame emphasizes the feelings of humiliation, inferiority, and inadequacy that come from seeing the self as defective (e.g., Tangney et al., 1996). Our conceptualization and operationalization of guilt do not include this sense of personal defect.

^2^Please note that the results were not altered when the covariate was removed. See Supplementary material for the direct and indirect effects of this model.

^3^It should be noted however that we tested an alternative mediational model reversing the causal ordering of emotions and moral conviction. We found no differences in the fit indices between the models, but we found a change in the indirect effect of RDBW on collective action through the sequential mediation of moral conviction and fear that become not significant, estimate = −0.010, *p* = 0.399, 95% CI [0.038, 0.010]. This result may indicate that the path model hypothesized in the study should be more adequate than the reversed. Of course, experimental research is needed to better clarify such an effect.
