# Supplementary material for: Men’s reactions to gender inequality in the workplace: From relative deprivation on behalf of women to collective action
Source: Front Psychol. 2022 Nov 17;13:999750. doi: 10.3389/fpsyg.2022.999750 (PMC9712440; doi:10.3389/fpsyg.2022.999750)
Supplement: Supplementary file 1 [file Table_1.doc]

**Men’s reactions to gender inequality in the workplace: From relative deprivation on behalf of women to collective action**

Silvia Mazzuca^1†*^, Silvia Moscatelli^1†^, Michela Menegatti^1^, Monica Rubini^1^

^1^Department of Psychology, Alma Mater Studiorum University of Bologna, Italy

^†^These authors contributed equally to this work and share first authorship

***Correspondence:**Corresponding Author silvia.mazzuca@unibo.it

**Supplementary material**

We hereby present the results of the hypothesized model without political orientation as covariate.

**Supplementary Figure 1**

Standardized solution of the model testing the relations among RDBW (relative deprivation on behalf of women), emotions, moral conviction, and collective action, with β (SE) and [95% CI].

.030

.44*** (.048) [.345, .531]

.27*** (.056) [.166, .383]

.28*** (.056) [.165, .383]

.25*** (.063) [.128, .375]

.39*** (.065) [.264, .519]

- .36*** (.056) [-.468, -.251]

- .28*** (.067)

[-.414, -.157]

.03 (.062)

[-.086, .155]

.20** (.061) [.079, .317]

Note. ***p* < .01; ****p* < .001

**Supplementary Table 1**

Total, total indirect, and specific indirect effects of relative deprivation on behalf of women (RDBW) on Collective Action

| Effects of RDBW on Collective Action | Estimate (SE) | [95% CI] |
| --- | --- | --- |
| Total | .50 (.047)  .30 (.039)  -.01 (.023)  .12 (.029)  .10 (.029)  .04 (.014)  .05 (.012) | [.457, .715] |
| Total indirect |  | [.246, .461] |
| RDBW 🡪 fear🡪 collective action |  | [-.070, .037] |
| RDBW 🡪 guilt🡪 collective action |  | [.080, .218] |
| RDBW 🡪 moral conviction🡪 collective action |  | [.052, .199] |
| RDBW 🡪fear🡪moral conviction🡪 collective action |  | [.021, .087] |
| RDBW🡪 guilt🡪moral conviction🡪 collective action |  | [.029, .091] |

The dataset presented in this study can be found in online repository at osf.io/q7kjs
